# Efficacy of a Novel Vertebral Body Augmentation System in the Treatment of Patients with Symptomatic Vertebral Body Fractures

**DOI:** 10.1007/s00270-020-02658-4

**Published:** 2020-10-25

**Authors:** Stefano Marcia, Emanuele Piras, Joshua A. Hirsch, Alessio Mereu, Mariangela Marras, Alessio Spinelli, Luca Saba

**Affiliations:** 1 UOC of Radiology, Ospedale SS Trinità, ATS, Cagliari, Italy; 2 Neuroendovascular Program, Department of Radiology, Massachusetts General Hospital, Harvard Medical School, Boston, MA USA; 3 Department of Radiology, Azienda Ospedaliero Universitaria (A.O.U.), Di Cagliari, Polo di Monserrato s.s. 554 Monserrato, 09045 Cagliari, Italy; 4 UOC of Radiology, Ospedale Brotzu, AOB, Cagliari, Italy

**Keywords:** Vertebral body augmentation, Vertebral body fractures, Osteoporosis

## Abstract

**Purpose:**

To evaluate the safety and efficacy of a novel augmentation implant in the treatment of patients with symptomatic vertebral body fractures.

**Materials and Methods:**

Thirty consecutive patients (seven males and 23 females), mean age of 70 years (range 56 to 89) with osteoporotic fractures and/or low-energy trauma fractures (osteoporosis confirmed by CT), were enrolled in an IRB-approved prospective study. The type of fracture was classified according to the Magerl classification. The patients were treated with the Tektona^®^ dedicated vertebral body augmentation system. Visual analogue scale (VAS) and Oswestry Disability Index (ODI) scores were obtained after 1, 6 and 12 months. Quality of life was assessed with the SF36 score.

**Results:**

A total of 37 vertebral bodies, mostly from T6 to L5, were treated in the 30 enrolled patients. In 67.6% of the cases (*n* = 25), lumbar fractures were treated. Most of the fractures (43%; *n* = 16) were A1.1 according to the Magerl classification. A significant pain reduction evaluated by VAS scores (*p* < 0.0001) was observed on average 7.6 (before the procedure) to 2.8 (immediately post-treatment), 2.1 and 2.7 (after 6 and 12 months later, respectively). The mean ODI score was 55.5% before treatment, and this was statistically significant reduced to 22.3% and 26.9%, respectively, at 6 and 12 months after treatment (*p* < 0.0001). The SF36 scores, both physical and mental components, showed statistically significant variations (*p* < 0.0001) whose direction was subpopulation dependent.

**Conclusion:**

Patients with confirmed osteoporosis, suffering from symptomatic vertebral body fractures (osteoporotic and/or low-energy traumatic), were treated safely and effectively using this novel implant.

## Introduction

The treatment strategy for thoracolumbar vertebral fractures ranges from non-operative to combined anterior and posterior stabilization [1–3]. There is uncertainty as to the benefits of operative vs. conservative therapy in patients with thoracolumbar burst fractures [4, 5]. However, significantly higher radiologic kyphosis and pain scores after non-operative treatment have been reported [6].

Treatment for patients with vertebral osteoporotic fractures and/or low-energy traumatic fractures comprises a wide spectrum of modalities including vertebroplasty (VP) and kyphoplasty (KP) [7]. More recently, implants and vertebral body stenting (VBS) have been introduced for the treatment of vertebral body fracture (VBF) [8]. The VBS technique, introduced in 2014 [8], uses an expandable metal stent to restore vertebral height. More complex implants have also been utilized for simultaneous multi-column stabilization. Over the years, techniques other than VBS have been introduced such as the titanium and polyetheretherketone (PEEK) implants which had previously been used in spinal surgery with low rejection rates [9].

This study presents the results from the initial experience utilizing a novel operative approach, the Tektona^®^ system. Safety and efficacy of the device and associated operative procedure rely on prospectively obtained validated outcome measures.

## Materials and Methods

### Study Design and Patient Population

This is a prospective, single-center, longitudinal study approved by the Institutional Ethics Review Board. From June 2015 to March 2016, thirty consecutive patients (seven males and 23 females) agreed to participate and were enrolled. The mean age was of 70.1 ± 7.8 years. These patients had painful osteoporotic fractures and low-energy traumatic fractures. Visual analogue scale (VAS) and Oswestry Disability Index (ODI) were recorded at baseline, 6 and 12 months after treatment; VAS was also checked immediately postoperatively (within 48 h). The diagnosis of osteoporosis was obtained through a dual-energy X-ray absorptiometry with a T score ≤ − 1.5.

Both CT and MRI were performed at baseline (the fracture was defined as acute or unhealed as demonstrated by T2 weighted STIR MRI) to fulfil the inclusion criteria. CT was performed immediately postoperatively and 12 months after treatment, to quantify variation in kyphosis, vertebral height and volume. Demographic data including age, weight and BMI were collected. Patient characteristics and baseline data are summarized in Table 1. Data were collected via phone calls, ambulatory visits and diagnostic examinations. Vertebral fractures were classified according to their Magerl [10] type and are described in Table 2. Total operation time, radiation dose, injected cement volume as well as any adverse events were recorded and are summarized in supplemental Table 1.

**Table 1:** Patient demographics and baseline characteristics

Characteristics	Value
Number of patients	30
Age (years) (mean ± SD, range)	70.1 ± 7.8 (56–89)
Gender (*N*, %)	
Male	7 (23.3%)
Female	23 (76.7%)
BMI (mean ± SD, range)	23.6 ± 3.5 (16–29)
Vertebral fracture (*N*, %)	
Osteoporosis	23 76.7%)
Trauma	7 (23.3%)
Elapsed time since fracture (*N*, %)	
0–4 weeks	12 (40%)
5–8 weeks	10 (33.3%)
8 + weeks	8 (26.7%)
Pain medication (*N*, %)	
WHO I	24 (80%)
WHO II	6 (20%)
Pre-operative status (mean ± SD, range)	
VAS	7.6 ± 1.6 (4–10)
ODI (%)	55.5 ± 18.8 (31.0–95.5)
SF36—physical component	40.7 ± 7.9 (28.5–59.4)
SF36—mental component	47.2 ± 11.4 (29.7–71.4)

**Table 2:** Spinal levels treated and Magerl’s classification

Treated level	Magerl’s classification				Total	
	A1.1	A1.2	A1.3	A2.1	*N*	%
Th6	–	2	–	–	2	5.4
Th7	–	2	–	–	2	5.4
Th8	1	–	–	–	1	2.7
Th9	1	–	–	–	1	2.7
Th10	–	1	–	–	1	2.7
Th11	–	–	1	–	1	2.7
Th12	1	2	–	1	4	10.8
L1	1	1	5	–	7	18.9
L2	2	–	1	–	3	8.1
L3	5	–	1	–	6	16.2
L4	4	1	1	–	6	16.2
L5	1	1	1	–	3	8.1
Overall	16 (43.3%)	10 (27.0%)	10 (27.0%)	1 (2.7%)	37 (100%)	100.0

### Primary and Secondary Objectives

#### Primary Objectives

1. *Safety of the procedure in terms of peri- and postoperative risk* (immediate, short and long term): (a) embolism, vascular and/or canal leakage; (b) neurological injuries; (c) subsequent fracture and/or adjacent body fracture; (d) device failure including lamellar fracture; (e) procedural blood loss; (f) major operative complications; (g) revisions and re-interventions.
2. *Efficacy*: (a) height restoration of treated vertebra, as evaluated by anterior, posterior and middle vertebral body heights (VBH); (b) kyphosis reduction at the treated level, as evaluated by vertebral kyphosis (VK) and local kyphosis (LK).

#### Secondary Objective

1. Assessment of the injected cement volume.
2. Pain control: self-evaluation by the patient using VAS, preoperatively, and at 6 and 12 months postoperatively.
3. Assessment of the health-related quality of life improvement and patient satisfaction (self-assessment with the SF36 survey): at 6 and 12 months, compared to the initial preoperative status; both the physical component (PC) and mental component (MC) were assessed.
4. Functional assessment (self-assessment by using ODI index): at 6 and 12 months, compared to the preoperative status.

### Inclusion and Exclusion Criteria

The inclusion criteria were based on the fracture type according to the cause, clinical status of the patient and imaging data. All patients had fulfilled the following conditions: (1) age > 18 years; (2) maximum two vertebral fractures from T5 to L5; (3) Magerl-type A1–A3; (4) painful fracture(s), dating from < 6 weeks; (5) ODI > 30; (6) failure of conservative treatment after 2 weeks or more; (7) imaging (CT and MR) showing; (8) vertebral kyphosis of ≥ 15° for thoracic and ≥ 10° for lumbar vertebral body fractures; (9) compression of ≥ 15° compared to adjacent vertebrae; (10) vertebral lateral cuneiform angle of at least 10°; and (11) hyperintense signal on T2 STIR MR images.

Exclusion criteria were defined as: (1) fracture consolidation (spontaneous healing with no pain); (2) concomitant neurological pathologies; (3) need of spine cord decompression; (4) vertebra plana; (5) posterior wall damage with neurological deficit; (6) local infection; (7) nerve compression pathology (fragments and/or compression); (8) anteroposterior spine canal reduction of more than 50%; (9) bleeding disorder (low platelets, high INR, hematological disease); (10) pregnancy; and (11) patient refusal to be enrolled in the study.

Follow-up the VAS postoperatively (at 48 h), 6 and 12 months time points after treatment was performed. A similar approach was applied for the Oswestry Disability Index with a 6- and 12-month assessment after treatment. CT assessment was performed postoperatively and at 12 months after treatment.

### The Tektona^®^ System

#### Description of the Device

**Fig. 1:**
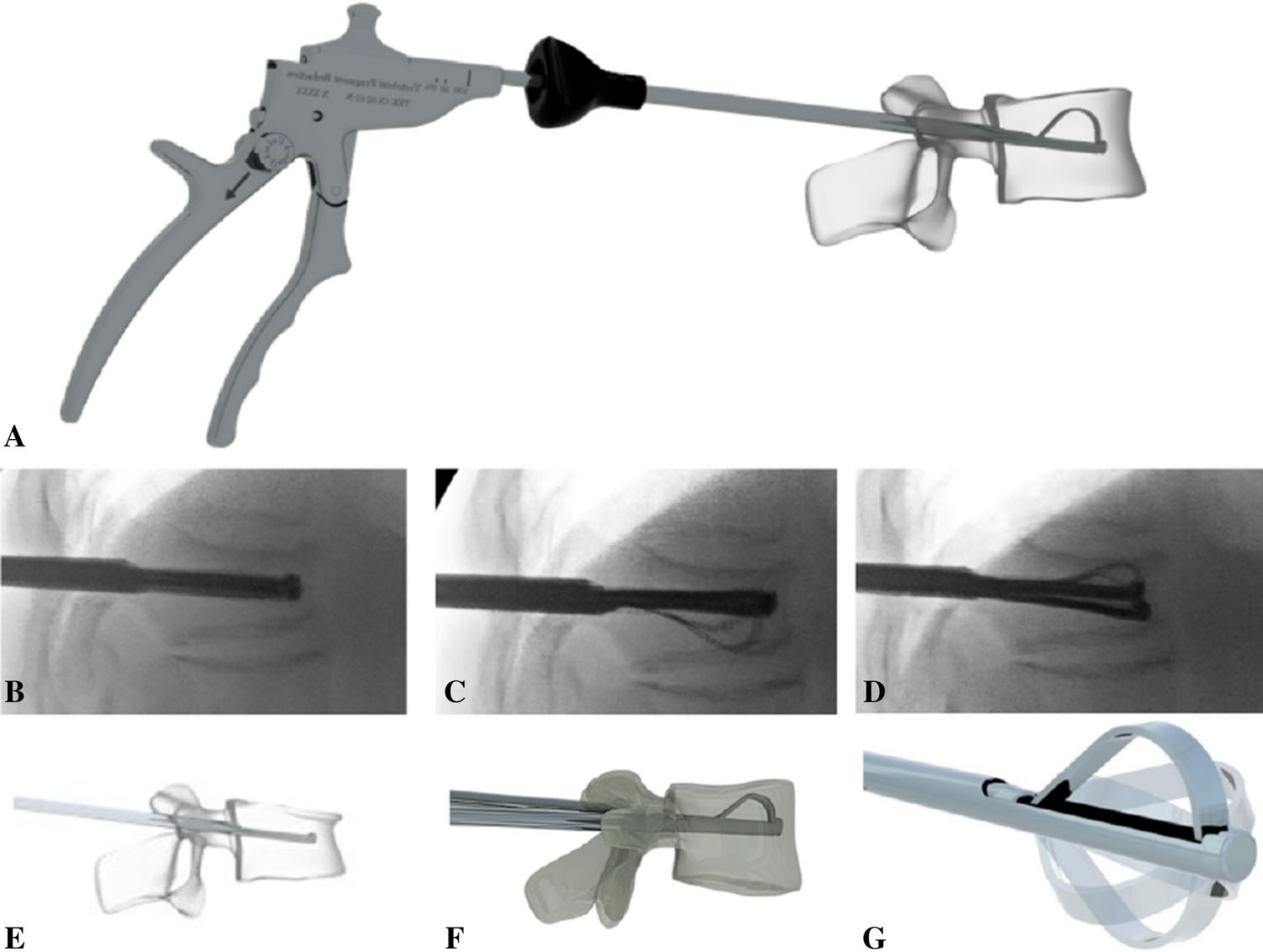
In this figure, the panel **a** shows the shape configuration of the Tektona system, whereas in the panels **b**–**d** the different phases of the placement are given under fluoroscopy and with the scheme (panels **e**–**g**). Illustration included with the permission of Spineart SA, Geneva

Tektona^®^ is a vertebral body augmentation system that assists in the reduction of vertebral body fractures, using a percutaneous, minimally invasive approach combined with injection of a dedicated bone cement (Fig. 1). According to the manufacturer’s IFU, the Tektona^®^ system is indicated to treat moderate to severe pain caused by vertebral body compression fractures (VCF) located between T7 and L5, presenting kyphotic deformities and risk of progressive vertebral height loss. It is approved for use in Europe.

The system consists of a flexible lamella (nitinol, a nickel–titanium alloy) which can be shaped by the action of a vertebral fragment reduction (VFR) instrument. The VFR instrument has a novel blocking system that can maintain the shape of the lamella during the fracture reduction procedure. The VFR instrument enables controlled expansion; it can be locked once expanded so as to maintain restoration, while expansion is carried out on the other pedicle. The 3D design theoretically should simultaneously afford controlled expansion capabilities while preserving the trabecular structure of the vertebrae. Nitinol provides enough flexibility for different elevation maneuvers to be carried out during vertebral fragment reduction. The instrument can be used to treat multiple levels.

#### Technique used for the Procedure

All procedures were performed with a fluoroscopic C arm (OEC 9900 Elite, GE OEC Medical Systems, USA). Periosteal injection was accomplished with 5 ml of subcutaneous 2% lidocaine [LidocaineChloridrate, Phisiopharma SH, Salerno, Italy] and 5 ml of 7.5% ropivacaine [RopivacaineChloridrate, Bioindustria LIM, Novi Ligure, Italy]. Deep sedation was given by the anesthesiologists and consisted of intravenous midazolam 0.05 mg/kg (Ipnovel, Roche, Basel, SWI) + propofol 1–4 mg/kg (B. Braun, Melsungen AG, Germany). Procedural monitoring included electrocardiogram, oxygen saturation and hemodynamic parameters. Two grams of ceftazidime (Tazidif, S.f. Group Srl, Rome, Italy) 30 min before the exam was administered to the patient.

**Fig. 2:**
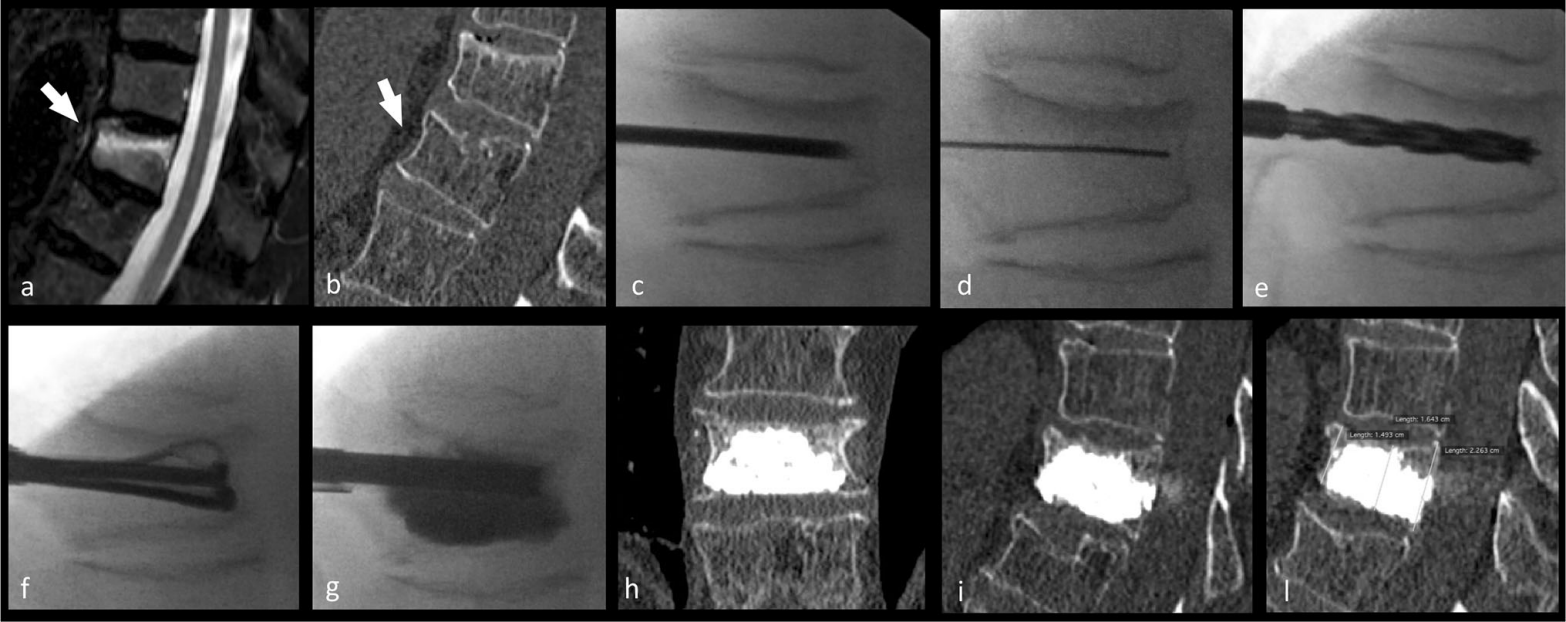
In this figure, the case of a 63-year-old female subject presented with acute back pain of 7 on the VAS. Panel **a** shows sagittal fat-saturation T2 weighted image and shows an A1.1 Magerl-type fracture at L2 (white arrow); Fig. 1b shows corresponding sagittal bone window CT image. In the panel **c**–**g**, the different phases of the Tektona bone remodeling procedure are given. In the panel **c**–**g**, the different phases of the Tektona bone remodeling procedure are given: **c** introduction of the trocar needle into the vertebral body; **d** the Kirschner wire has been introduced and the trocar needle removed; **e** the drill has been advanced over the Kirschner wire; **f** insertion of the VFR device and expansion of the lamella; **g** after the VFR device has been totally removed, the bone fillers were introduced and the bone cement has been injected. In the panel **h** and **i**, the postoperatively acquired CT is given, whereas in the panel **l**, the 12-month follow-up

All procedures were performed utilizing a bipedicular approach: 11-gauge needles were inserted using the oblique view and then advanced in the anteroposterior (AP) projection to the medial aspect of the pedicle. The technique of bone augmentation was performed using standardized technique (Fig. 2) [11]. The maximum possible expansion avoids damaging the interbody plate: The mechanism allows a controlled expansion.

The PMMA (Mendec^®^ Spine HV System, Tecres, ITA) injection was done under continuous fluoroscopic guidance to avoid leakages. The desired endpoint for cement injection was when the spreading of the PMMA covers from superior to inferior end plate. The working time of this cement is about 15 min. Then, the bone fillers and the working cannulas were removed. After the procedure, the patients were kept for three hours in hospital for clinical monitoring of their recovery and then discharged.

### CT Measurements

CT exams were performed with a Somaton 40 Scanner (Siemens; Erlangen, Germany) with volumetric acquisition by using a pitch of 1, slice thickness 1 mm, FOV 150 mm and kernel B70s (very sharp). Exams were performed just before and after the procedure and at 12-month follow-up, and quantitative evaluations were performed for vertebral height and kyphosis.

*Height of the vertebral body* was acquired in three different locations: anterior vertebral body height (anterior VBH), posterior vertebral body height (posterior VBH) and mid-vertebral body height (middle VBH).

The *Kyphosis* was assessed as:

- (a) *Vertebral kyphosis* (VK): the angle between the superior and inferior plate of the treated vertebra and
- (b) *Local kyphosis* (LK) as the Cobb angle: angle between the superior end plate one level above the treated vertebra and the inferior end plate one level below the treated vertebra (Fig. 2).

The *volume of the vertebral body* was calculated using dedicated software (Osirix 7.0, PixMeo). Two experienced neuroradiologists, blinded to the technique, reviewed the CT and the Magerl-type classification.

### Statistical Analysis

Descriptive statistics methodology was mainly used in this study. Categorical data were presented as contingency tables. The normality of each continuous variable group was tested using the Kolmogorov–Smirnov *Z* test. For normal distributed values, data were described as the mean value ± SD, whereas for non-Gaussian distributions, the median, range and interquartile range (IQR) values were given. The Wilcoxon test was applied to compare preoperative and postoperative values and test the statistical significance of differences in VAS and ODI scores, in vertebral heights, kyphosis angles and vertebral volumes. The statistical significance level was set at 95% (*p* ≤ 0.05). The *SAS* V 9.4 software was used for statistical analyses.

## Results

### General and Anatomical Results

Thirty consecutive patients were enrolled and treated at one or two levels. The total number of treated levels was 37 as seven patients underwent a simultaneous two-level treatment. A total of 12 thoracic and 25 lumbar fractures were treated. According to the Magerl classification, there were 16 A1.1-, ten A1.2-, ten A1.3- and one A2.1-type fractures. All the procedures were performed exclusively on T6 to L5 spinal levels. Twenty-five lumbar levels were treated (67.6%).

Most of the fractures (*n* = 16) were A1.1 according to Magerl type (43% of the total). The incidence of treated levels and VCFs type according to Magerl’s classification is presented in Table 2. The volume of injected PMMA was 4.2 ± 0.7 ml (range 3–6 ml) per treated vertebra. The total operation time (from the local anesthesia to the end of the procedure, after the bone cement was injected) was 30.7 ± 7.6 min (range 18–60 min), and the fluoroscopic exposure time was 7.1 ± 1.5 min (from 4.7 to 10 min). All 30 patients (37 levels) underwent 6-month follow-up, while 26 patients (30 levels) also underwent 12-month follow-up.

**Fig. 3:**
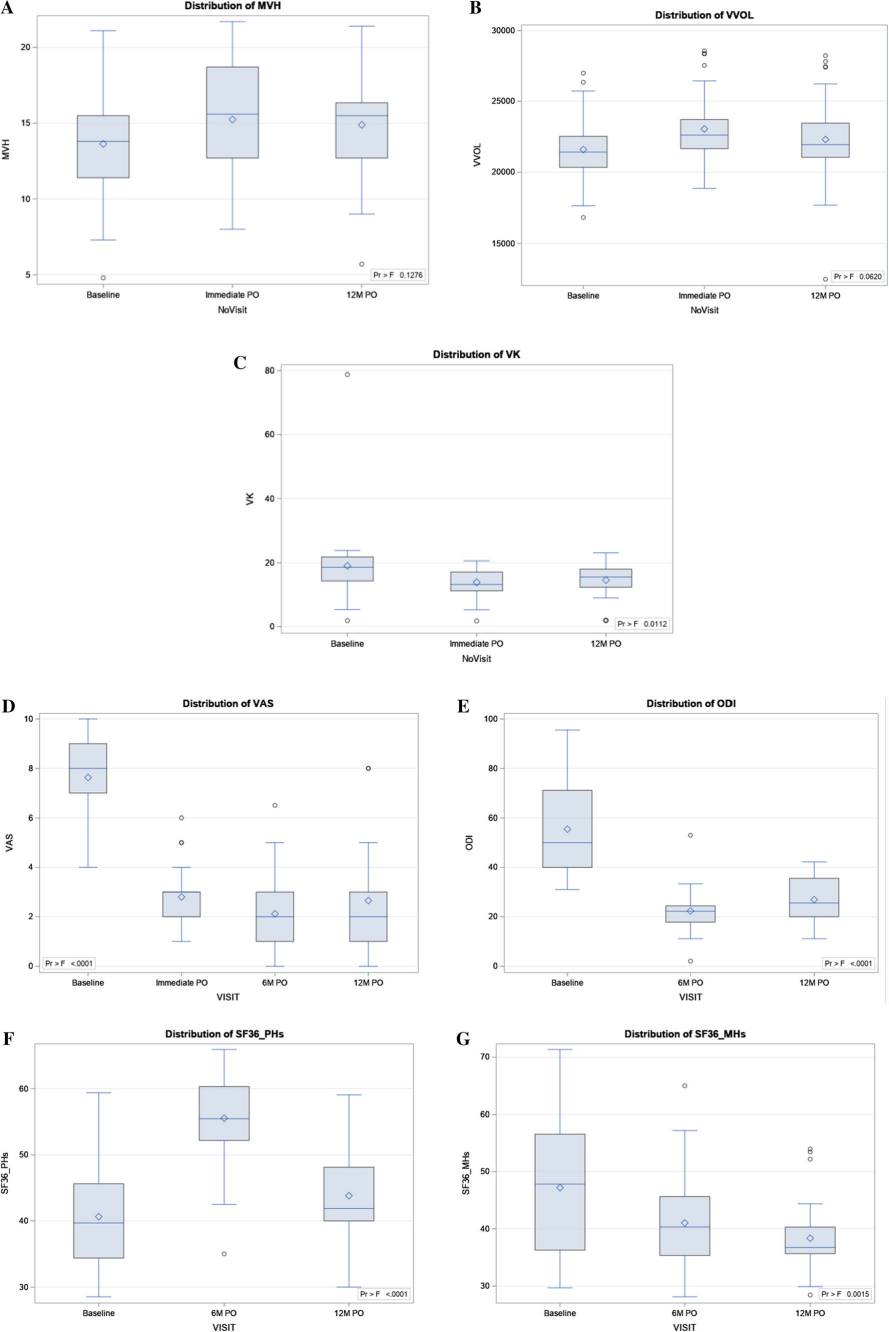
Boxplot of middle vertebral height: pre- and postoperative value (**a**), boxplot of the vertebral body volume: pre- and postoperative values (**b**), boxplot of the vertebral kyphosis: pre- and postoperative values (**c**); boxplot of the VAS index: pre- and postoperative values (**d**); boxplot of the ODI scores: pre- and postoperative values (**e**); boxplot of the SF36 physical health score: pre- and postoperative values (**f**); boxplot of the SF36 mental health score: pre- and postoperative values (**g**). *Maximum* is the endpoint of upper whisker, *third quartile* (75th percentile/P75) is the upper edge of box, *median* (50th percentile) is the line inside box, *mean* is the symbol marker, *first quartile* (25th percentile/P25) is the lower edge of box and *minimum* is the endpoint of lower whisker

The efficacy was evaluated by the (positive) variation in vertebral body heights (anterior, middle and posterior) and by the reduction (if any) of kyphotic angles. Results are summarized in Table 2 and shown in Fig. 3. Height restoration of treated vertebrae, as evaluated by anterior, posterior and middle VBH, showed a significant improvement. The middle vertebral height (MVH) was restored immediately postoperatively in average with 1.61 mm, and the anterior vertebral height (AVH) observed a mean variation of 1.0 mm.

Kyphosis reduction at the treated level, as evaluated by VK immediately postoperatively, showed statistically significant values, in average, a reduction of 5.1°.

The volume of the treated vertebral bodies showed a significant increase immediately postoperatively (*p* = 0.001), in average, 1.5 cm^3^ as compared to its pre-op value (Table 3).

**Table 3:** Comparison of means between pre- and postoperative vertebral parameters

Time point	*N*	Mean ± SD	Comparison		Difference between means		95% CI	
							Min.	Max.
Anterior vertebral height (AVH) (mm)								
Pre-op	37	17.6 ± 4.2	–		∆	*p* value*		
Imm PO	37	18.6 ± 4.3	Pre-op		0.9919	0.001	− 1.3058	3.2896
12 M PO	32	17.7 ± 4.0	Pre-op		0.0968	0.009	− 2.2889	2.4825
Middle vertebral height (MVH) (mm)								
Pre-op	37	13.6 ± 3.6	–		∆	*p* value		
Imm PO	37	15.2 ± 3.5	Pre-op		1.6111	0.001	− 0.3441	3.5663
12 M PO	32	14.9 ± 3.5	Pre-op		1.2555	0.001	− 0.7746	3.2856
Posterior vertebral height PVH (mm)								
Pre-op	37	21.1 ± 3.5			∆	*p* value		
Imm PO	37	22.1 ± 3.8	Pre-op		1.0405	0.001	− 1.0193	3.1004
12 M PO	32	21.5 ± 3.8	Pre-op		0.4476	0.001	− 1.6912	2.5864
Vertebral volume (VVOL) (cm^3^)								
Pre-op	37	21.6 ± 2.3			∆	*p* value		
Imm PO	37	23.1 ± 2.4	Pre-op		1.4612	0.001	0.0073	2.9151
12 M PO	32	22.3 ± 3.1	Pre-op		0.7081	0.012	− 0.8015	2.2177
Local kyphosis (LK) (°)								
Pre-op	37	12.2 ± 4.7			∆	*p* value		
Imm PO	37	10.0 ± 4.1		Pre-op	− 2.228	0.001	− 4.699	0.243
12 M PO	32	10.8 ± 4.5		Pre-op	− 1.457	0.005	− 4.023	1.109
Vertebral kyphosis (VK) (°)								
Pre-op	37	19.0 ± 11.3			∆	*p* value		
Imm PO	37	13.9 ± 4.4		Pre-op	− 5.133	0.001	− 9.430	− 0.837
12 M PO	32	14.6 ± 5.4		Pre-op	− 4.448	0.001	− 8.909	0.013

^*^p values from Wilcoxon analysis

### Clinical Results

Pain control, self-evaluated by the patient using the VAS, was on average 7.6 before the procedure and fell statistically significant (*p* < 0.0001) to 2.8, 2.1 and 2.7, respectively, for immediate post-op, 6 and 12 months later (Table 4, Fig. 3).

**Table 4:** Patient’s self-reported parameters: summary statistics of pre- and postoperative values

Parameter	Time point	N	Mean	SD	Median	Min.	IQR		Max.
							P25	P75	
VAS	Pre-op	30	7.6	1.6	8.0	4.0	7.0	9.0	10.0
	Imm post-op	30	2.8	1.1	3.0	1.0	2.0	3.0	6.0
	6 M PO	30	2.1	1.4	2.0	0.0	1.0	3.0	6.5
	12 M PO	26	2.7	2.1	2.0	0.0	1.0	3.0	8.0
ODI	Pre-op	30	55.5	18.8	50.0	31.0	40.0	71.1	95.6
	6 M PO	30	22.3	8.4	22.2	2.0	17.8	24.4	53.0
	12 M PO	26	26.9	9.5	25.6	11.1	20.0	35.6	42.2
SF36 PHs	Pre-op	30	40.6	7.9	39.7	28.5	34.4	45.6	59.4
	6 M PO	30	55.6	6.6	55.5	35.0	52.2	60.3	65.9
	12 M PO	26	43.8	6.6	41.9	30.0	40.0	48.1	59.1
SF36 MHs	Pre-op	30	47.2	11.4	47.8	29.7	36.3	56.6	71.4
	6 M PO	30	41.0	8.4	40.3	28.1	35.3	45.6	65.0
	12 M PO	26	38.3	6.7	36.7	28.4	35.6	40.3	54.0

Functional disability, as evaluated by the ODI scores, was in average 55.5% before the treatment and decreased (*p* < 0.0001) to 22.3% at 6 months and 26.9% at 12 months after treatment (Table 4, Fig. 3).

Overall patient satisfaction, self-evaluated using the SF36 scores, showed statistically significant improvement in the mental category at both 6 and 12 months post-op (*p* = 0.015 and 0.002, respectively); the physical category also showed a statistically significant difference at 6 months (*p* = 0.001), whereas no significant difference was found at 12 months (Table 4, Fig. 3).

There were no severe or major complications (embolism; neurological injuries, blood losses; surgery major complications). Two cases of vascular leakages and six cases of discal cement extrusion have been observed. All leakages were asymptomatic at all time points. There were no epidural leaks. No system breaking (lamella) was observed. Eight new fractures (five adjacent and three non-adjacent) were observed at 6-month follow-up controls (all were retreated with percutaneous vertebroplasty), while no new fractures were observed at 12 months.

## Discussion

Symptomatic osteoporotic vertebral body fractures represent a critical public health problem due to the large number of people suffering from this condition and the high associated direct and indirect costs of this pathology [12, 13]. In particular when conservative treatment is ineffective, the surgical approaches are subject to questions about high costs and peri/post-procedural risks [14, 15]. Therefore, the introduction of efficient, minimally invasive, techniques to treat this condition brought important direct benefit both to the patients and the healthcare system. The first suggested such systems were vertebroplasty (VP) and kyphoplasty (KP) with good safety profiles as well as very good results in terms of pain reduction and functionality [16, 17]. In particular, KP aims at restoring the height of the vertebra, in contrast to VP, which primarily seeks biomechanical stability and pain relief, through injection of bone cement [18, 19]. In this scenario of clinical uncertainty, using appropriateness method (such as the RAND/UCLA) may be helpful to support decision making in daily clinical practice and to improve quality of care and for the indication of the best therapeutic strategies [20].

Over the last few years, several vertebral augmentation (VA) devices have been introduced as an evolution of KP, specifically aiming at the remodeling of the vertebral body, in order to reach the best long-term height restoration. It is thus currently possible to use a variety of dedicated tools [21–23]. Among these vertebral augmentation devices, the Vertebral Body Stenting^®^ (VBS) (Synthes, Soletta, Switzerland) consists of balloon expandable stents delivered into the vertebral body as an internal cast *before* the injection of the bone cement. The OsseoFix^®^ (Alphatec Spine Inc., Carlsbad, CA) consists of an intra-vertebral mesh cage implant that can be introduced with or without PMMA injection [24, 25]. The SpineJack (Vexim, SA, Balma, France) showed superiority to KP in restoring vertebral heights in a single-center trial and in a cadaver study [8, 9, 26]. Another VA technique is the KIVA (Benvenue Medical Inc., Santa Clara, CA) system, which uses a flexible implant made of a medical polymer to restore height to the vertebral body and hold the cement [27].

In a single-center randomized trial comparing KP with VBS, Werner et al. [28] reported that no neurological or other severe complications were detected and that no surgical revision was needed for the VBS arm. The present study suggests that this technique could be better with respect to cement extravasation, as compared to their reported values of 20% for minor leakages and up to 10% for major ones [28]. Additionally, four material-related complications were described in the same study (a balloon rupture and three stent ruptures). Higher values of leakages (39%) were reported by Noriega et al. [29] using the SpineJack. In the KAST trial [27], a total of 300 subjects with one or two painful osteoporotic VCFs were randomized to blindly receive Kiva (*n* = 153) or BK (*n* = 147) and they were followed through 12 months. The authors found that the Kiva system is noninferior to BK based on a composite primary endpoint assessment incorporating pain-, function- and device-related serious adverse events for the treatment of VCFs due to osteoporosis.

In the present study, there were a total of eight new fractures (21.6% of the initial number) at 6-month follow up: five at adjacent levels (13.5%) and three at non-adjacent (8.1%). All were symptomatic and treated with percutaneous vertebroplasty. No additional new fracture was observed at 12-month follow up. These results are higher than those reported in the literature with different devices: With the use of VBS, the risk of adjacent vertebral fracture was 9% [26] and 3% with SpineJack [29]. In these studies, the cohorts of patients were similar and these were performed on osteoporotic subjects with spontaneous fractures or minor trauma in osteoporosis.

In the SAKOS trial [30], it was demonstrated the noninferiority of the SpineJack compared to balloon kyphoplasty. Both techniques were shown to display very good clinical efficiency and safety with comparable effects on the improvement in daily functions and quality of life. SpineJack showed better results in terms of pain relief, midline VB height restoration and incidence of adjacent fractures.

The volume of the treated vertebral bodies showed a significant increase immediately postoperatively (*p* = 0.001), on average, 1.5 cubic cm as compared to its pre-op value, but there was a decrease in volume and angulation at 12 months. This finding is similar to what occurs with the other augmentation systems where a postoperative increase is documented followed by a reduction approaches after 6–12 months. This could be explained perhaps by the fact that the filling of the bone is never completely from upper plate to lower plate and the fact that the vertebrae are malacic contributes to the partial loss of the height gained.

As detailed previously, efficacy results were good with regard to the chosen endpoints: Height restoration of treated vertebrae, kyphosis reduction at the treated level and volume increase in the treated vertebral bodies were all shown to improve significantly. Similarly, self-evaluations by the patients of their pain level (VAS), their disability index (ODI) and their global satisfaction (SF36) indicated clear improvements in these aspects both at 6 and 12 months after treatment. It is interesting to note that the ODI decreased from 55.5 to 22.3 at 6 months and then rose slightly to 26.9% at 12 months. This slight worsening of the patients between the 6- and 12-month endpoint could be explained by the fact that when patients do not undergo new fractures, they often suffer from concomitant degenerative phenomena and therefore they have a back pain for other causes; moreover, muscle trophism is usually reduced with less biomechanical support and also worsens in one year in elderly patients.

The Tektona^®^ is different from other implants such as VBS or SpineJack [30], being made up of an expandable device which is removed at the end of the procedure, unlike the others which are intra-somatic prostheses. Moreover, compared to the intra-somatic prostheses, Tektona^®^ has lower costs and it is possible to treat multiple levels with the same device. Therefore, in multilevel osteoporotic patients this could have economic benefit. It is important to underline that the less expansive force is compared with other systems such as the SpineJack but is usually enough in osteoporotic patients.

There are limitations to this study: The number of patients enrolled is small (*n* = 30) and all procedures were performed by an experienced operator in a single center. The small number of patients could lead to poor reproducibility of the results. Multi-center studies with a larger population will be helpful in confirming these results. Additionally, the inclusion of two patients having two VBF treated in the same procedure could introduce heterogeneity in the study population: Thoracic and lumbar vertebral body fractures do not behave identically, which could determine a bias; moreover, treating two levels: one in the thoracic and one in lumbar, may alter mechanics of an osteoporotic spine. Another limitation of this study is that there was no control group. Finally, the single center is considered to be quite experienced in this domain; this might indicate that there are limitations in the generalizability of these results, particularly in terms of the prevalence of procedure-related complications.

## Conclusion

Symptomatic acute osteoporotic compression fracture treated with the Tektona vertebral body augmentation device achieved significant reduction in pain scores and disability measures. The procedural complication rate was low.
